# Granulysin expressed in a humanized mouse model induces apoptotic cell death and suppresses tumorigenicity

**DOI:** 10.18632/oncotarget.11473

**Published:** 2016-08-22

**Authors:** Ya-Wen Hsiao, Tsung-Ching Lai, Yu-Hsiang Lin, Chia-Yi Su, Jih-Jong Lee, Albert Taiching Liao, Yuan-Feng Lin, Shu-Chen Hsieh, Alexander T.H. Wu, Michael Hsiao

**Affiliations:** ^1^ Genomics Research Center, Academia Sinica, Taipei, Taiwan; ^2^ Department of Obstetrics and Gynecology, Chung Shan Medical University Hospital, Taichung, Taiwan; ^3^ School of Veterinary Medicine, National Taiwan University, Taipei, Taiwan; ^4^ Graduate Institute of Clinical Medicine, College of Medicine, Taipei Medical University, Taipei, Taiwan; ^5^ Institute of Food Science and Technology, National Taiwan University, Taipei, Taiwan; ^6^ Ph.D. Program for Translational Medicine, College of Medical Sciences and Technology, Taipei Medical University, Taipei, Taiwan; ^7^ Department of Biochemistry, College of Medicine, Kaohsiung Medical University, Kaohsiung, Taiwan

**Keywords:** granulysin, humanized mouse model, apoptosis, tumorigenicity

## Abstract

Granulysin (GNLY) is a cytolytic and proinflammatory protein expressed in activated human cytotoxic T lymphocytes (CTLs) and natural killer (NK) cells. Conventional mouse models cannot adequately address the triggering mechanism and immunopathological pathways in GNLY-associated diseases due to lack of the GNLY gene in the mouse genome. Therefore, we generated a humanized immune system (HIS) mouse model by transplanting human umbilical cord blood mononuclear cells into NOD.Cg-*Prkdc^scid^ Il2rg^tm1Wjl^*/SzJ (NSG) mice after sublethally irradiation. We examined the GNLY expression and its effects on tumor growth using this system. Our HIS mice expressed human CD45^+^, CD4^+^, CD8^+^ and CD56^+^ cells in the peripheral blood and spleen. A high expression level of human Th1/Th2 and NK cytokines was detected, indicating the activation of both T and NK cells. Importantly, we found an elevated level of GNLY in the serum and it was produced by human CTLs and NK cells obtained from the peripheral blood mononuclear cells and spleen cells in the HIS mice. The serum level of GNLY was negatively correlated with the proliferation of transplanted tumor cells in HIS mice. Collectively, our findings strongly supported that HIS mouse as a valuable model for studying human cancer under an intact immune system and the role of GNLY in tumorigenesis.

## INTRODUCTION

Granulysin (GNLY), a member of the saposin-like protein (SAPLIP) family, is produced by activated human cytotoxic T lymphocytes (CTLs) and natural killer (NK) cells [1, 2]. GNLY exhibits a broad spectrum of antimicrobial activities and potent cytotoxic action against tumor cells [3, 4]. GNLY protein is shown to activate antigen presenting cells and serve as immune alarmin [5]. In addition, GNLY acts as a chemoattractant for T cells, monocytes, and other inflammatory cells and to stimulate the expression of several cytokines, including RANTES, IL-1, IL-6, IL-10, and IFN-γ [6], indicating GNLY helps recruit immune cells to sites of inflammation. GNLY has been shown as a lytic agent against various human tumors [4, 7] and may be useful as a diagnostic and therapeutic agent in clinics [8]. Experimentally, GNLY promoted survival in transgenic mice with tumor [9]. GNLY expression level was correlated with the prognosis and progression of cancer patients [8, 10, 11]. Unfortunately, the conventional mouse model cannot be used to study the triggering mechanism and immunopathological pathways mediated by GNLY due to lack of the GNLY gene in the mouse genome [6, 9].

Mice with humanized immune system (HIS) is a more physiological equivalent to the traditional immunocompromised mouse system which is a standard for examining a variety of human disorders, particularly for cancer. HIS serves as a powerful preclinical model for examining human infections, cancer, transplantation and immune-mediated diseases as well as with the functional roles of GNLY in these processes. A successful humanized mouse model will not only reveal the pathological mechanism of the diseases, but also provide a useful preclinical research tool for evaluating the efficacy of therapeutic interventions in an individualized fashion. Therefore, we generated a HIS mouse model by intravenously injecting human umbilical cord blood mononuclear cells (CBMCs) into sublethally irradiated NOD.Cg-*Prkdc^scid^ Il2rg^tm1Wjl^*/SzJ (NSG) mice [12] and examined the GNLY expression and its role in tumorigenesis. First, we established a HIS model where human CD45^+^, CD4^+^, CD8^+^ and CD56^+^ cells and human Th1/Th2 cytokines were detected. Next, we demonstrated that GNLY was present in the serum and splenocytes; notably, GNLY was produced by CTLs and NK cells of human origin in our HIS mice. Importantly, GNLY down-regulated the tumor growth via apoptotic pathways and the serum level of GNLY was correlated with the inhibition of tumorigenesis in HIS mice. We believe that our GNLY-expressing HIS mouse model could be a valuable platform for exploring a variety of biological processes and therapeutic development for personalized medicine.

## RESULTS

### Phenotypic characterization of the NSG mice hematopoietic system

**Figure 1:**
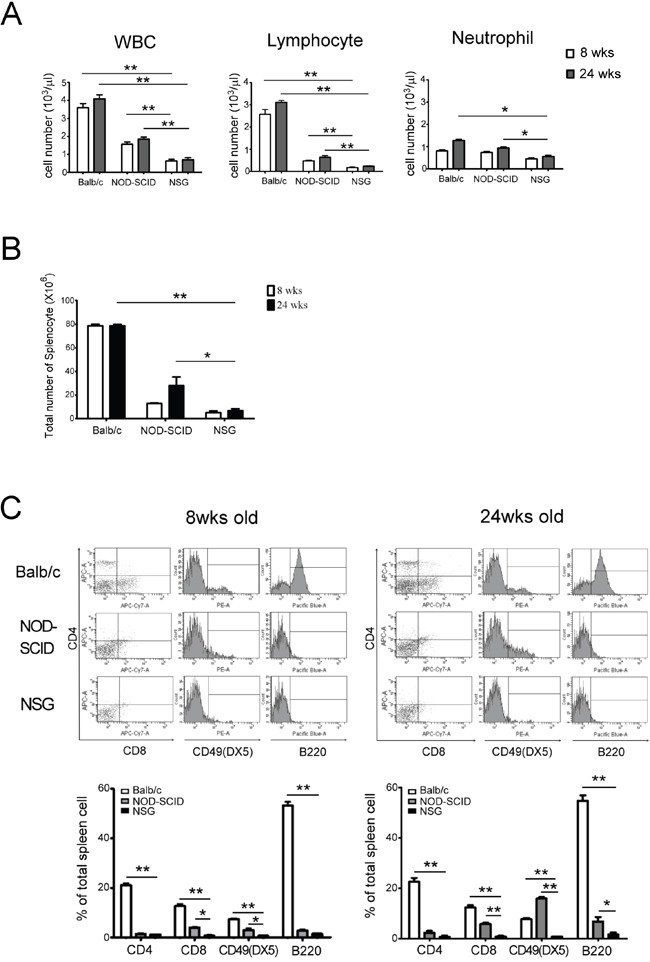
Characteristic of hematology and spleen cell populations in 8 and 24 wks old Balb/c, NOD-SCID and NSG mice **A.** There is a significantly few in peripheral leukocyte counts in NSG mice compared with Balb/c and NOD-SCID. The lymphocyte counts in NSG are substantially lower than in Balb/c and NOD-SCID. In NSG mice, the lymphocyte counts showed no noteworthy difference at 8 and 24 wks old resulting from no leakiness. **B.** The total splenocyte in NSG mice was lower than the other two strains. The number of splenocyte between NOD-SCID and NSG was significantly different at 24 wks old. **C.** The expressions of CTL and NK cells in NSG were significantly lower than in Balb/c and NOD-SCID mice at 8 and 24 wks old. The leakiness of NK cells were found in old NOD-SCID but not in NSG mice. Each value represents the mean ±SD of three or more repeats experiments. (^*^ p<0.05, ^**^ p <0.01)

Prior to the engraftment of human cord blood mononuclear cells (CBMCs), the hematological profiles of the peripheral blood obtained from naïve male Balb/c, NOD-SCID and NSG mice (8 and 24 weeks of age) were examined and compared. The numbers of white blood cells (WBC), neutrophils and lymphocytes were significantly lower in the NSG mice as compared to those in Balb/c and NOD-SCID mice. There was no significant difference in lymphocyte and neutrophil counts between 8 and 24 week old NSG mice. This suggested that aging process did not change the lymphocyte counts in NSG mice resulting from no leakiness (Figure 1A). At 24 weeks, the number of splenocytes in the NSG mice (7.03×10^6^ ±0.25) was 10- and 4-fold less as compared to those in Balb/c (7.8×10^7^±0.25; p<0.01) and NOD-SCID (2.8×10^7^±1.53; p<0.05) mice respectively (Figure 1B). IL2Rγ- chain is required for the development and function of hematopoietic cells. We then examined the effects of the loss of IL2Rγ- chain, particular in NK cell that constrains human CBMC engraftment in NSG mice. T, B and NK cell numbers were significantly lower in NSG mice as compared to those in Balb/c and NOD-SCID mice (Figure 1C, p<0.05). The leakiness of NK cells was found in old NOD-SCID but not in NSG mice (Figure 1C) suggesting NSG mouse as a more receptive model for establishing the human immune system.

### Detection of human CBMC in NSG mice using biofluorescence imaging

**Figure 2:**
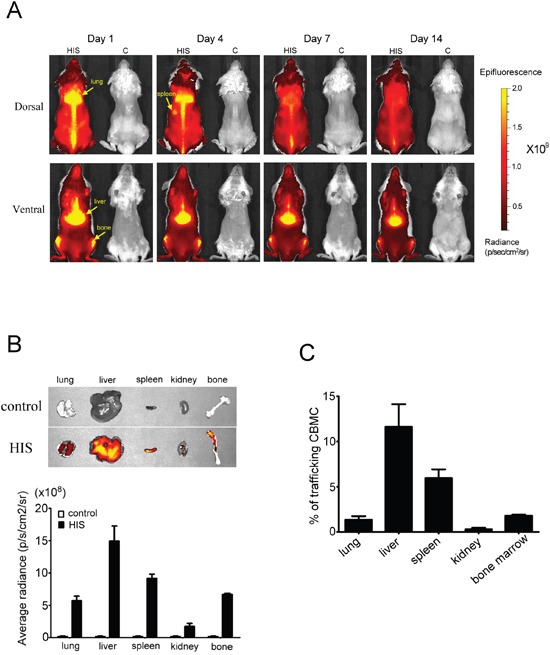
*In vivo* biofluorescence images of human CBMC trafficking in NSG mice Human CBMCs were stained with xenolight-DiR dye and then injected into sublethal irradiated NSG mice through the tail vein. The control mice were injected free dye only. **A.** From the *in vivo* images, we found the human CBMCs were successful transplantation in NSG mice (left side in each photo) and might mainly distribute in liver, spleen, lung and femur. **B.** On day 14, the mice were sacrificed and then quantitated the fluorescence intensity from lung, spleen, liver, kidney and femur by IVIS. The average radiance in liver and spleen was higher than the other organs. **C.** The single suspension cells from these organs were used to analyze the percentage of trafficking CBMCs by FACSCanto to confirm the cells distribution. Each value represents the mean ±SD of three or more repeats experiments.

To monitor human CBMC engraftment in NSG mice, biofluorescence imaging was performed using CBMCs that were stained with xenolight-DiR dye [13]. CBMCs stained with DiR dye and free dye only were intravenously injected into sublethally irradiated NSG mice. CBMCs were mainly distributed in the liver, spleen, femur and lungs (Figure 2A). The mice were sacrificed two weeks post transplantation and the detection and quantification of CBMCs was determined from the lungs, liver, spleen, kidney and femur. CBMCs were mainly detected in the liver and spleen as indicated by intense fluorescent signals and less found in the lung, kidney and bone marrow (Figure 2B). Cells isolated from these organs were analyzed cytometrically and reconfirmed our imaging results (Figure 2C). Collectively, our data demonstrated the human CBMCs were engrafted successfully in NSG mice and trafficking to liver, spleen and bone marrow.

### Phenotypic analysis of human lymphocytes and examination of human cytokine production in human CBMC transplanted NSG mice

**Figure 3:**
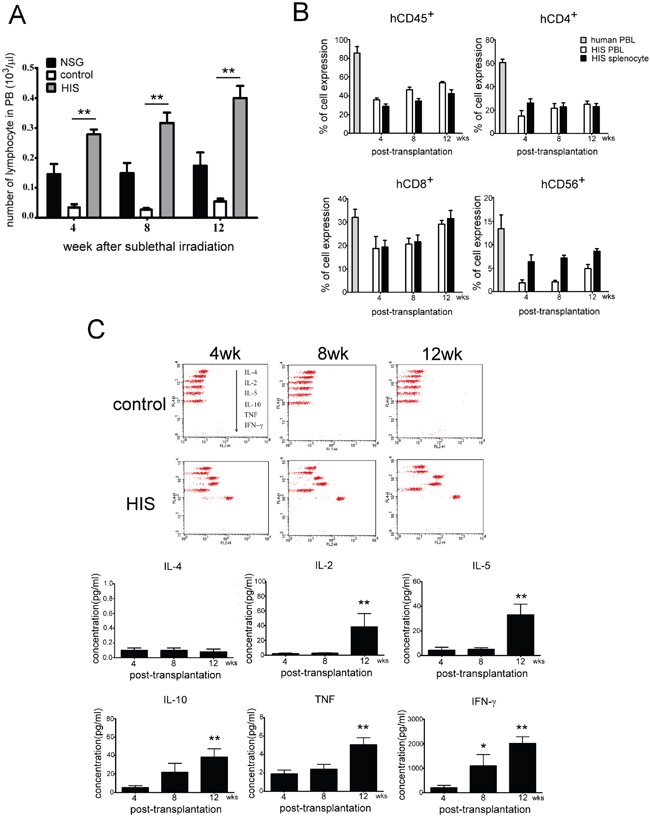
Analysis of lymphocyte phenotype and human cytokines production after human CBMC transplantation in NSG mice **A.** The numbers of lymphocyte in peripheral blood of normal NSG, control (with TBI) and HIS (TBI plus human CBMC engraftment) mice were evaluated. The control mouse lost almost all lymphocytes in peripheral blood and did not recover as the normal NSG. Human CBMC engraftment gradually reconstructed lymphocytes population of peripheral blood in HIS mouse. (■ normal NSG; □ control; ■ HIS). **B.** Phenotypes of human lymphocytes were detected subsequently in PBL and spleen of HIS mice. The population of human CD45^+^ cells was increased gradually in PBL and spleen, whereas the human CD4^+^ cells stayed low till 12 weeks after CBMC transplantation. The expressions of human CD8^+^ and CD56^+^ cells were similar to the human at 12 weeks. **C.** Th1/Th2 and NK cytokine levels were also monitored at 4^th^, 8^th^ and 12^th^ week after CBMC engraftment. The level of IL-2, IL-5, IL-10, TNF and IFN-γ was increase along with time, particularly at 12^th^ week. Each value represents the mean ±SD of three or more repeats experiments.

After establishing that CBMC was successfully transplanted into NSG mice, we evaluated the number of lymphocytes in peripheral blood obtained from different NSG mice groups namely, the normal NSG, control (received total body irradiation, TBI) and HIS (TBI plus human CBMC engraftment). The control mice were found almost depleted with all lymphocytes in peripheral blood and did not recover as in the normal NSG mice. Comparatively, a significant and gradual increase in the number of lymphocytes were observed in the HIS mice (Figure 3A). We then evaluated the efficiency of human CBMC engraftment and determined the development of human lymphocytes in NSG mice. Using flow cytometric method, we detected PBLs and splenocytes positive for human CD45^+^ expression 4 weeks post CBMC engraftment, and the percentage of human CD45^+^ cells increased gradually overtime (Figure 3B), suggesting the HIS mouse model was established successfully. Furthermore, we showed that human T and NK cells were found in the PBL and spleen of NSG mice. The number of human CD8^+^ and CD56^+^ cells increased 12 weeks post CBMC transplantation in NSG and the expression profiles were comparable to that of the human (Figure 3B). To determine the activation of lymphocyte in HIS mice, Th1/Th2 and NK cell cytokine levels were monitored at 4^th^, 8^th^ and 12^th^ week after CBMC engraftment. The level of IL-2, IL-5, IL-10, TNF and IFN-γ increased over time, particularly at 12^th^ week (Figure 3C). Nevertheless, IL-4 was undetectable in the serum of HIS mice.

### Expression of GNLY in HIS mouse

**Figure 4:**
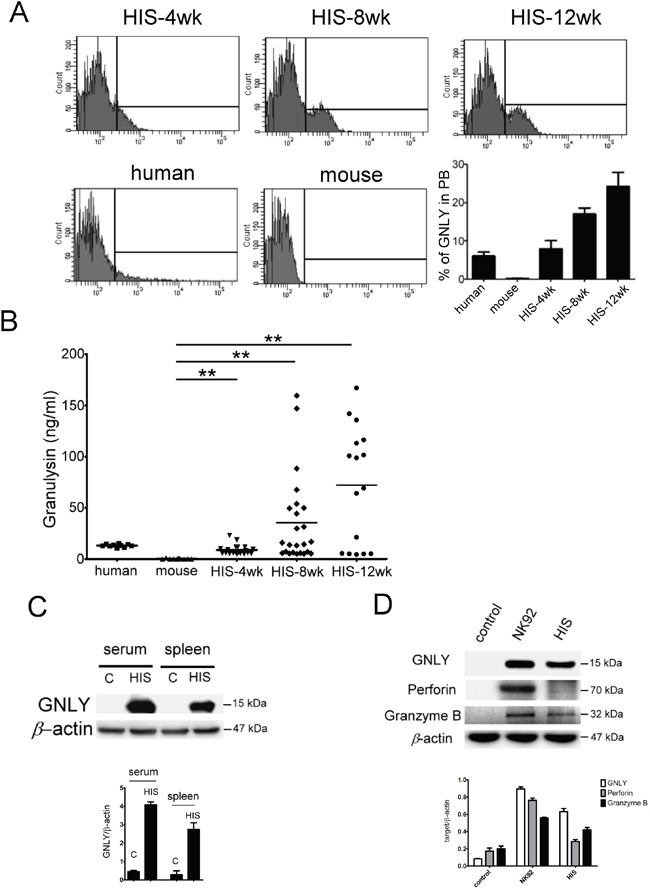
Expression of GNLY in HIS mice **A.** The intracellular GNLY was detected in PBL of HIS mouse, healthy human and NSG mouse by FACS analysis. The level of GNLY expression at 4^th^ week after CBMC transplantation was similar to the healthy human and increased gradually later. **B.** The concentration of GNLY from HIS mouse serum was demonstrated by ELISA. Amounts of GNLY protein were expressed in serum and the level of GNLY was increased gradually after CBMC transplantation in HIS mice. Expression of GNLY was not found in the mouse. **C.** GNLY protein was found in serum and splenocytes of HIS mice. **D.** Western blot of GNLY, perforin and granzyme B in splenocytes of HIS mice. Each value represents the mean ±SD of three or more repeats experiments.

**Figure 5:**
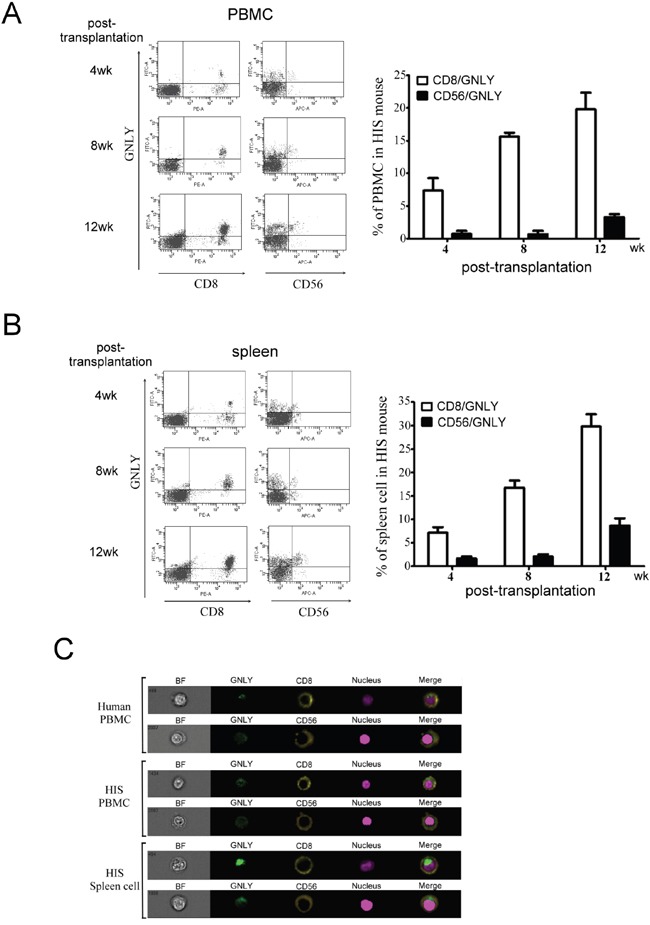
GNLY was produced by human CTL and NK cells of PBMC and spleen in HIS mice CTL and NK cells collected from **A.** PBMC and **B.** spleen of HIS mice was used to measure GNLY expression by FACS analysis. **C.** The single cell image of double staining of GNLY with either CTL or NK cell marker was measured by ImageStream multispectral imaging flow cytometer. Each figure is representative example of at least three independent experiments with similar results.

GNLY is a cytolytic protein present in the granules of activated human CTLs and NK cells but not in the mice. Four weeks after CBMC transplantation, the GNLY expression was found in HIS mice (Figure 4A). The serum concentration of GNLY in the HIS mice increased gradually and the level was similar to a healthy human 4 weeks post CBMC transplantation (Figure 4B); this phenomenon was not observed in the control counterparts. The amount of GNLY was found higher in the serum than in splenocytes (Figure 4C). GNLY has been detected in human CTL and NK cells along with perforin and granzyme B. In our study, the expression of GNLY was more than perforin and granzyme B in the splenocytes of HIS mice (Figure 4D). Using FACS analysis, we demonstrated that GNLY was produced by human CTLs and NK cells of PBMC and the spleen from HIS mice (Figure 5A, 5B). The single-cell images of human CTL and NK cells expressing GNLY (in cytoplasmic granules) were captured and analyzed by ImageStream multispectral imaging flow cytometer (Figure 5C).

### GNLY inhibited tumor growth through the apoptosis pathway in HIS mice

**Figure 6:**
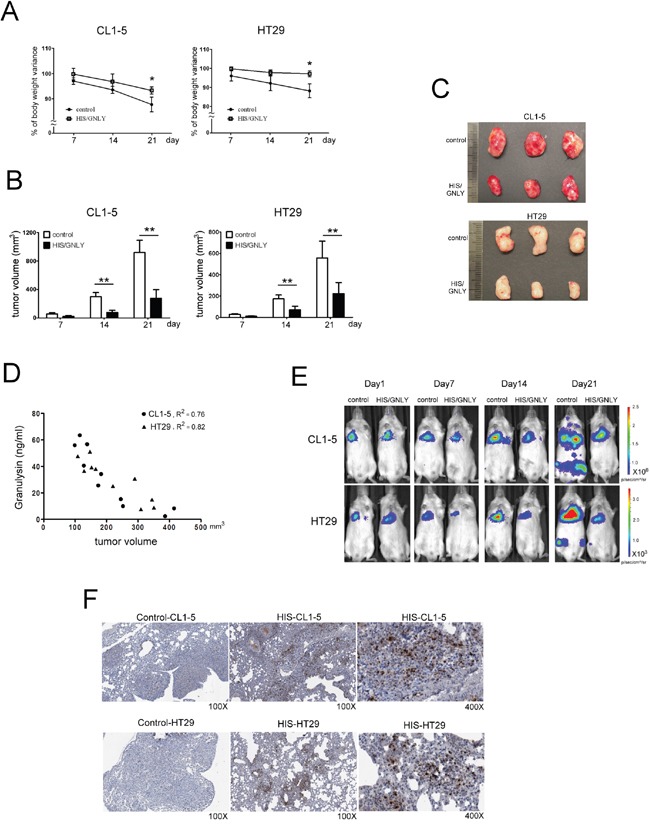
Tumor growth was inhibited in GNLY expressed HIS mice **A.** Variations of body weight were monitored weekly after tumor injection. At day 21, the body weight was decreased significantly in control mouse compare to HIS mice **B.** 5×10^6^ CL1-5 and HT29 tumor cells were inoculated subcutaneously on HIS mice with GNLY expression (n=10) and control (n=10), respectively. Tumor volume was measured weekly by caliper. Since day 14, the size of tumor masses on HIS mouse was significantly smaller than those on control mouse. (p<0.01) **C.** The photos presented tumor masses collected from HIS mice and control at day 21 after tumor injection. **D.** The level of GNLY from tumor-bearing HIS mice serum were measured by ELASA to study the relation with tumor volume. **E.** HIS (n=7) and control mice (n=7) were challenged with tail vein injection of 1×10^6^ CL1-5 and HT29 tumor cells, respectively. The *in vivo* images were monitored weekly by IVIS. **F.** In immunohistochemistry staining, amounts of GNLY were infiltrating in tumor section of HIS mice that were challenged with tail vein injection of tumor cells. (^*^ p<0.05, ^**^ p <0.01)

**Figure 7:**
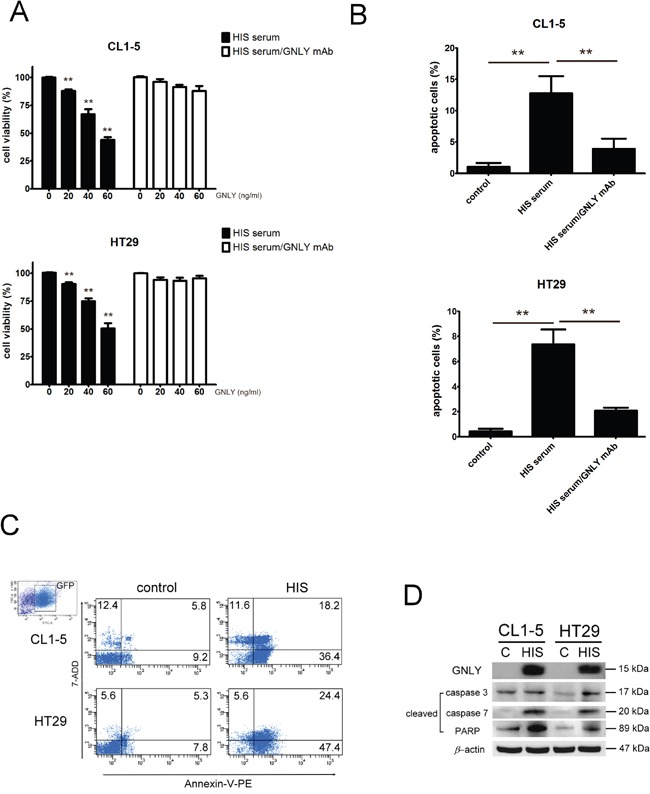
GNLY inhibited tumor growth through apoptosis pathway **A.** CL1-5 and HT29 tumor cells were cultured with various concentration of GNLY (evaluated by ELISA) from the serum of HIS mice. For blocking GNLY experiment, GNLY mAb was incubated with tumor cells to neutralize the GNLY before serum treatment. The viability of tumor cells was measured by alamar blue reduction assay **B.** Tumor cells were treated with 50 ng/ml GNLY from the HIS mice serum and the apoptosis was measured by Annexin V-PE/7-ADD double staining flow cytometry. **C.** The tumor cells were isolated from the lung of HIS and control mice and apoptosis was performed by Annexin V-PE/7-ADD double staining flow cytometry. **D.** Western blot of GNLY, cleaved caspase3, caspase7 and PARP from the tumors of HIS and control mice. Each value represents the mean ±SD of three or more repeats experiments.

To examine the potential tumorcidial effects of GNLY, HIS and control (standard NOD/SCID mice) were challenged with subcutaneous injection of 5×10^6^ CL1-5 (metastatic lung cancer cells) and HT29 (colorectal cancer cells) tumor cells. The tumor volume was significantly smaller in HIS mice at day 14 (p<0.01, Figure 6B, 6C) The body wieght of both control and HIS mice bearing CL-1-5 tumor cells decreased signficantly 21 days after tumor injection (Figure 6A); In HT29 group, the control mice showed a significant decrease in body weight (Figure 6A). Importantly, we found that the serum level of GNLY correlated negatively to the tumor volume in both CL1-5 and HT29-bearing HIS mice (p<0.01, Figure 6D). Parallelly, in a metastatic xenograft model, CL1-5 and HT29 (1×10^6^ cells/injection) tumor cells were intravenously injected into HIS and control mice. The bioluminescence imaging indicated a significant decreased tumor burden and metastasis in HIS animals as compared to the control mice in both CL1-5 and HT29 groups (Figure 6E). Immunohistochemical analysis of the tumor samples demonstrated that a more intense GNLY staining in HIS mice than ones in control mice (Figure 6F). In addition, GNLY obtained from HIS mice serum suppressed the viability of tumor cells (Figure 7A) and induced apoptosis (Figure 7B). The tumor cells were not inhibited when the effect of GNLY was neutralized. Comparative flowcytometric analysis was performed on tumor cells obtained from HIS and control mice. A significantly higher number of CL1-5 (18.2% vs. 5.8%) and HT29 (24.4% vs 5.3%) tumor cells were double-labelled by Annexin-V and 7-ADD in HIS samples as compared to control samples (Figure 7C). Western blots of these samples showed GNLY expression along with an increased expression of cleaved caspase3, caspase7 and PARP in the HIS mice (Figure 7D). Together these observations suggested that GNLY was produced by human CTLs and NK cells and GNLY mediated anti-tumor effects via the induction of caspase-dependent apoptosis in HIS mice.

## DISCUSSION

GNLY is important in a variety of human diseases, including infections, [14] transplantation, [15] and cancer [4, 16, 17]. The physiological function of GNLY has been mostly derived from *in vitro* studies [3, 18, 19] and clinical correlations, [11, 20, 21] anti-microbial being its major function [5, 7, 22]. Although the mouse model has been a valuable *in vivo* tool for examining human diseases, however, mice lack GNLY gene. Thus, the conventional mouse model cannot be used to study the underlying mechanisms and immunopathological signaling involved in GNLY associated diseases. In this study, we established the HIS mice that expressed GNLY which is produced by engrafted human CTLs and NK cells. Our HIS mouse model represents a valuable platform for studying the role of GNLY-associated biological functions and process.

HIS mouse is now an important tool for studying of the pathogenesis of human-specific diseases *in vivo* [23]. The well-established NOD-SCID mice have been widely used as efficient recipients for reconstitution of human hematopoietic stem cells and human cancer xenografts. However, our data indicated that NOD-SCID mice contain a higher number of residual CTLs and NK cells than NSG mice and exhibit lymphocyte leakiness at old age. To improve engraftment efficient, the recent breakthrough in the development of humanized mice is the generation of targeted knockout of interleukin 2 receptor γ-chain (IL2Rγ) gene. The deficiency of IL2Rγ gene may lead to severe innate immunity defects to result in a complete loss of NK cells, which are a major obstacle for the engraftment of human hematopoietic stem cells and PBMCs [23–27]. Our data indicated a significantly low number of CTLs and NK cells in NSG mice demonstrated no leakiness of NK cells in old NSG mice. We have successfully generated a HIS mouse model from CBMCs transplantation into irradiated NSG mice, indicated by the presence of human CD45^+^, CD8^+^ and CD56^+^ cells in the peripheral blood and spleen at 4^th^ week post-transplantation. In addition, we demonstrated the level of GNLY was detected in HIS mouse serum and produced by human CTLs and NK cells.

Since the GNLY homologs are not found in mouse, transgenic mouse was also engineered to assess the *in vivo* effects of GNLY [6, 16, 28] Huang et al., [28] demonstrated that NK cells of GNLY^+/-^ transgenic mice only expressed the 15 kDa form of GNLY and a high level of both 9 and 15kDa forms of GNLY was found when activated by IL-15. Unlike the transgenic mouse study, GNLY secreted by human CTLs and NK cells in our HIS model was verified and found in the serum and spleen. We found that a higher level of GNLY in the serum than in the splenocytes. GNLY is known to be synthesized as a 15kDa precursor form, which subsequently is sorted to the cytolytic granules and processed into a 9kDa effector form [29]. Previous studies indicated that the recombinant 9kDa GNLY is broadly antimicrobial [3, 30] and is tumoricidal. The 9kDa GNLY in normal serum is hardly detected and even in serum from patient with acute primary EBV infection [31]. It is possible that the 9kDa GNLY might be rapidly adsorbed to membrane lipids in the neighborhood cells as well as to extracellular matrix because of its strong positive charge [32]. Intradermal injection of recombinant 15 kDa GNLY led to skin necrosis and blisters in skin that mimicks the clinical features of both Stevens-Johnson syndrome and toxic epidermal necrolysis [33]. Park et al. have shown that 15 kDa granulysin may have beneficial prognostic significance after pretreatment of the patients with diffuse large B cell lymphoma [10]. Thus, our HIS mouse model which express GNLY could be valuable for exploring the function(s) of GNLY *in vivo*.

GNLY has been shown to be expressed by the activated CTLs and NK cells and is found in the cytolytic granules with perforin and granzymes [3]. From our single cell imaging study, double staining of GNLY with either human CD8^+^ or CD56^+^ cells, indicated the GNLY verified that it was expressed by CTLs and NK cells. Furthermore, a high level of human Th1/Th2 and NK cytokines was also detected in the HIS mouse serum suggesting the activation of CTLs and NK cells. The elevated level of Th1-type cytokines, IL-2 and IFN- γ at 12^th^ week post CBMC transplantation could be correlated to the activation of CTLs and NK cells.

Previous studies suggested the anti-tumor function of GNLY in cancer patients [7, 8]. Sekiya et al. [34] showed that GNLY as an effective therapeutic agent against small-cell lung cancer in mouse by inducing apoptosis of tumor cells. Pages et al. [35] found GNLY among other markers in infiltrating cells in colorectal cancer. In addition, these findings strongly suggest that GNLY plays an important role in antitumor immunity and may be a valid prognostic indicator. Orthotopic implantation is more realistic than subcutaneous tumor models. The subcutaneous environment precludes tumor cells from metastasizing, however it’s easy to measure the volume of tumor mass [36, 37]. In our studies, we demonstrated that HIS mouse expressed GNLY and its presence suppressed the growth of both CL1-5 and HT29 tumor cells as evident by the increased expression of GNLY within the tumor mass. We have also transplanted the tumor cells to observe the growth and metastasis by tail vein injection. The patient-derived orthotopic tumor model may be more suitable for clinical implication and will be pursued in the near future [36, 37]. Equally important, the addition of GNLY isolated from HIS mouse serum induced apoptosis in both CL1-5 and HT29 tumor cells. Lung tumor biopsies obtained from HIS mice showed increased cleaved caspase 3, 7 and PARA expression, indicating the apoptotic function of GNLY in both CL1-5 and HT29 cancer cells.

Many studies suggest GNLY has potential as a useful diagnostic biomarker and a new therapeutic for a wide variety of human diseases. To our knowledge, this study is the first investigation of the tumoricidal effects mediated by GNLY in a HIS mouse model. In addition, tumor cells trafficking in blood vessels are an important route for metastasis. To observe the tumor-host cell interactions in tumor microenvironment, the HIS model could have been much more useful if genetic fluorescent reporters of different colors were applied to color-code the human immune cells and tumor cells [38, 39]. Our HIS mouse model expressing GNLY would be a valuable tool for the development of more effective therapies and delineation of GNLY’s role(s) in a spectrum of human diseases.

## MATERIALS AND METHODS

### Mice and conditioning regimen

NOD.Cg-*Prkdc^scid^* *Il2rg^tm1Wjl^*/Sz (NSG) and NOD-SCID mice were obtained from Jackson Laboratory (Bar Harbor, ME, USA) and bred in animal facility of genomic research center, Academia Sinica. Balb/c mice were purchased from BioLasco (Taiwan Co., Ltd). All mice were used 6 to 8 weeks old and housed in microisolator cages, given autoclaved food and water and maintained under specific pathogen-free mouse room at 21±2 °C with 14:10 h light: dark cycle. Mouse studies were approved by Academia Sinica Institutional animal Care and Use Committee (AS-IACUC). Male NSG mice received sublethal total body irradiation (TBI) (300 cGy), using a RS 2000 X-ray biological irradiator, between 2 to 8 hours prior to the human umbilical cord blood mononeuclear cells (CBMC) injection. Control mice were irradiated but did not received CBMC.

### Human umbilical cord blood cell isolation and transplantation

Human umbilical cord blood (CB) was obtained from protocols approved by Chung Shan Medical University Hospital institutional review board. Cord blood mononuclear cells (CBMC) were isolated by using density centrifugation method with Ficoll-Paque™ PREMIUM (GE Healthcare). The isolated cells were washed twice, counted, and resuspended in phosphate-buffered saline (PBS). Cell suspensions containing 0.5-1×10^7^ CBMC in 0.1 ml PBS were injected intravenously via the tail vein in the irradiated NSG mice.

### Noninvasive fluorescence image

Human CBMC trafficking in NSG recipients was assessed noninvasively by fluorescence with IVIS spectrum (Xenogen, Alameda, CA, USA) as described [40]. CBMCs were incubated with 3.5μg/ml 1, 1’-dioctadecyl- 3, 3, 3’, 3’-tetramethylindotricarbocyanine iodide (DiR) dye for 30 min at 37 °C [13]. Cells were washed twice with PBS and the viability was verified by trypan blue staining method. Afterwards, the labeled cells were injected intravenously into NSG mice. Imaging was performed on Day1, 4, 7 and 14 after CBMC injection. The excitation and emission filter set in the IVIS was 710 to 760 nm [13]. Data was processed using the Living Image 3.1 software (Xenogen-Caliper) after drawing regions of interests of identical size around individual wells. Fluorescence imaging activity was quantified as average radiance (p/s/cm^2^/sr).

### Flow cytometry and ImageStream multispectral imaging cytometry analysis

APC anti-mouse CD4 (clone RM4-5), APC-Cy™7 anti-mouse CD8a (clone 53-6.7), PE anti-mouse CD49b/Pan-NK Cells (clone DX5), Pacific Blue™ anti-mouse CD45R/B220 (clone RA3-6B2),, PE-Cy™7 anti-human CD45 (clone HI30), APC anti-human CD56 (B159), PE anti-human CD8 (clone RPA-T8), APC-Cy™7 anti-human CD4 (clone RPA-T4) and Alexa Fluor®488 anti-human granulysin (clone RB1) were purchased from BD Pharmingen™. Single-cell suspensions from the peripheral blood lymphocyte (PBL) and spleen were labeled with fluorochrome-conjugated monoclonal antibodies (mAb) specific for human or mouse surface marker at 4°C for 1h. The samples labeled with human mAbs were fixation and permeabilization (Cytofix/Cytoperm, SD Pharmingen) and then stained with anti-GNLY mAb. Flow cytometric analysis was done by using a FACS Canto (BD Biosciences) and data were analyzed using BD software. The single cell images of double staining with CD8^+^ CTL with GNLY and CD56^+^ NK cells with GNLY were subjected to ImageStream multispectral imaging flow cytometer analysis (Amnis, Seattle, WA) and the IDEAS software package.

### Measurement of human cytokines

The serum samples were arranged from blood collected at 4^th^, 8^th^ and 12^th^ week after CBMCs transplantation. HIS mouse serum was analyzed for human Tumor Necrosis Factor (TNF), IL-2, IL-4, IL-5, IL-10, and Interferon-γ (IFN-γ) by using human Th1/Th2 Cytokine Cytometric Bead Array system (BD Bioscience). Following acquisition of sample data by using the flow cytometer, the results are then generated with a graphical and tabular format by using the BD FCAP Array^TM^ Software.

### Western blotting

The samples were separated on a 4-12% NuPAGE® Bis-Tris gel in a MES buffer system (Invitrogen) and the separated proteins were transferred to PVDF membrane (Millipore). The membrane was blocked in 5% BSA buffer for 1 h and then incubated with anti-GNLY mAb (clone RF10, MBL), anti-perforin Ab (abcam), anti-granzyme B Ab (BD pharmingen), anti-cleaved caspase3, caspase7 and PARA (Cell Signaling) mAb at 4°Covernight. Proteins were detected with Immobilon™ Western chemiluminescent HRP substrate (Millipore)

### Tumor challenge

The level of GNLY in HIS mice was confirmed by ELISA before tumor cells injection. HIS (at week 8) and control mice that no phenotype expressed were challenged with subcutaneous injection of 5×10^6^ stable CL1-5-GL and HT29-GL tumor cells. The tumor volume (volume = 1/2(length × width^2^)) and body weight were monitored weekly after tumor injection. Moreover, HIS and control mice were challenged with tail vein injection of 1×10^6^ stable CL1-5-GL and HT29-GL tumor cells. The luciferase expression and the concentration of GNLY from serum in HIS and control mice were monitored weekly by IVIS and ELISA, respectively after tumor injection. All mice were sacrificed when the body weight decreased by 20% in control mice to comply with the rules of Academia Sinica Institutional Animal Care and Use Committee (AS-IACUC). The lung from HIS and control mice were harvested and analyzed the GNLY expression by IHC stain.

### Cell viability assay

CL1-5 and HT29 tumor cells were seeded into quadruple wells of a 96-well plate containing 200 μL/well of 2.5 × 10^4^ to 1 × 10^6^ cells/ml concentration at a standard culture condition of 37°C with 5% CO_2_ in air. After 4 h incubation period to allow for cell attachment, 20, 40 and 60 ng/ml GNLY, evaluated by ELISA, from serum of HIS mice was treated with tumor cells for 2h. For blocking GNLY experiment, the 15 μg GNLY mAb (DH10, BioLegend®) was incubated with tumor cells for 30 minutes at 37°C to neutralized the GNLY before serum treatment. Then, 20 μl AlamarBlue® (AB) solution was directly added to the fresh medium to result in a final concentration of 10%. The plate was then returned to the incubator. The absorbance of test and control wells was read with a standard spectrophotometer at 540 and 630 nm 4h after adding AB. As a negative control, AB was added to medium without cells. AB reduction was calculated as previously described [41].

### Apoptosis assay

Tumors cells that were incubation with GNLY from HIS mice serum or isolated from tumor-bearing HIS mice were performed by staining annexin-V-PE and 7-ADD (BD Biosciences) according to the manufacturer’s instructions and then analyzed for apoptosis by flow cytometry. The tumor cells isolated from HIS mice were analyzed the expressions of cleaved caspase3, caspase7 and PARA by western blotting.

### Statistical analysis

The results are shown as individual data or as the mean ± SD. Statistical comparisons were performed according to Student’s t test. Three or more means were compared by using one-way analyses of the variance. The *P* value of < 0.05 was considered statistically significant. All other additional analyses were performed by using GraphPad Prism software (graphpad software, San Diego, CA, USA).
